# Association of sonographic estimated fetal weight with primary cesarean delivery after adjustment for neonatal birthweight in term nulliparous pregnancies

**DOI:** 10.3389/fmed.2026.1989181

**Published:** 2026-10-22

**Authors:** Ba-Nha Pham, Quang-Chien Chu, Van-Anh Nguyen, Phuong-Trang Dang, Thanh-Tung Do, Manh-Tin Doan, Phuong-Tu Nguyen

**Affiliations:** 1 Vinmec Times City International Hospital, Hanoi, Vietnam; 2 VinUniversity, Hanoi, Vietnam; 3 Hanoi Medical University, Hanoi, Vietnam; 4 Bach Mai Hospital, Hanoi, Vietnam

**Keywords:** birthweight, cesarean delivery, estimated fetal weight, nulliparity, obstetric ultrasound, term pregnancy

## Abstract

**Objective:**

To examine whether a relatively higher prenatal sonographic estimated fetal weight (EFW), compared with the newborn's eventual birthweight, is associated with primary cesarean delivery among term nulliparous pregnancies.

**Methods:**

We conducted a single-center retrospective cohort study at Vinmec Times City International Hospital between January and September 2024. Eligible women were nulliparous with singleton, cephalic, term pregnancies, entered spontaneous labor or underwent induction of labor, and had sonographic EFW recorded within 7 days of delivery. The linear association between EFW and neonatal birthweight was described using Pearson correlation, while agreement was assessed using signed error, mean absolute percentage error (MAPE), the proportion within ±10% of birthweight, and Bland-Altman analysis. The primary modified Poisson model examined EFW–birthweight discrepancy while accounting for neonatal birthweight and adjusting for maternal age, gestational age at delivery, fetal sex, and scan-to-delivery interval.

**Results:**

Among 353 women, 168 (47.6%) underwent cesarean delivery. Mean EFW was 3,205 ± 267 g and mean neonatal birthweight was 3,178 ± 314 g. Prenatal EFW and neonatal birthweight were positively correlated (Pearson r = 0.78). Mean signed error was +27.1 g, MAPE was 5.15%, and 89.0% of estimates were within ±10% of birthweight. In the primary linear model, each 100-g greater EFW–birthweight discrepancy was associated with an 11% higher risk of cesarean delivery after accounting for neonatal birthweight and prespecified covariates (adjusted risk ratio [aRR] 1.11, 95% CI 1.04–1.20; *p* = 0.003). Findings were consistent across prespecified sensitivity analyses (aRR range, 1.11–1.14).

**Conclusion:**

Among term nulliparous women, a relatively higher prenatal sonographic EFW compared with eventual neonatal birthweight was associated with a higher risk of primary cesarean delivery. This association should not be interpreted as evidence that knowledge of EFW caused cesarean delivery and should be considered in light of residual confounding and individual-level uncertainty in EFW.

## 1 Introduction

Cesarean delivery is common in obstetric practice but is associated with maternal risks and complications in subsequent pregnancies (1, 2). Safe reduction of primary cesarean delivery, particularly among nulliparous, term, singleton, cephalic pregnancies, is therefore an important goal of obstetric care (3).

Sonographic estimated fetal weight (EFW) is routinely used to assess fetal size and identify fetuses that are small for gestational age or large for gestational age (LGA) (4). LGA describes fetal or neonatal size relative to gestational age, whereas macrosomia refers to a high absolute fetal or neonatal weight (4). EFW is inherently an estimate, and measurement error may occur in either direction and may vary with fetal size (5). Consequently, prenatal EFW may differ substantially from eventual neonatal birthweight even in the absence of any change in clinical management.

A separate issue is whether knowledge or suspicion of a large fetal size influences obstetric management. Prenatal concern for LGA or macrosomia may affect counseling, decisions regarding induction or trial of labor, and thresholds for intrapartum intervention (6, 7). Several studies suggest that prenatal assessment of fetal size may be associated with cesarean delivery even after neonatal birthweight is considered. Dude et al. reported that, among nulliparous women with medication-requiring gestational or pregestational diabetes undergoing a trial of labor, the availability of a recent sonographic EFW was associated with a higher likelihood of intrapartum cesarean delivery even after adjustment for birthweight (8). Cooley et al. found that a third-trimester ultrasound diagnosis of LGA was associated with a higher likelihood of cesarean delivery, including among nulliparous, term, singleton, cephalic pregnancies; overall, the positive predictive value of the antenatal LGA diagnosis was approximately 50% (9). Sgayer et al. found that among newborns with an actual birthweight ≥4,000 g, cesarean delivery remained more frequent when macrosomia had been suspected prenatally (10). Lopian et al. reported a higher risk of intrapartum cesarean delivery among pregnancies screening positive for LGA or macrosomia, including false-positive cases (6). This possible management or ‘labeling’ effect is different from ultrasound measurement error.

Previous studies differ substantially in study population, exposure definition, and the interval between ultrasound and delivery. Many have focused on categorical diagnoses of macrosomia or LGA or on selected high-risk populations, whereas fewer have modeled prenatal EFW continuously while explicitly accounting for eventual neonatal birthweight in a homogeneous cohort of term nulliparous women. We used a 7-day scan-to-delivery window to reduce differences caused by continued fetal growth while retaining most term records. We hypothesized that a relatively higher prenatal EFW compared with eventual neonatal birthweight would be associated with a higher risk of primary cesarean delivery. We therefore evaluated this association and additionally characterized agreement between prenatal EFW and neonatal birthweight.

## 2 Materials and methods

### 2.1 Study population

We conducted a single-center retrospective cohort study at the Department of Obstetrics and Gynecology, Vinmec Times City International Hospital, using routinely collected clinical data from electronic medical records. The source population included all women who delivered at Vinmec Times City International Hospital between January and September 2024 and had a singleton, cephalic pregnancy, available prenatal ultrasound biometry, and recorded neonatal birthweight.

To define the primary analytic cohort, we further required nulliparity, term gestation (37 + 0 to 41 + 6 weeks), spontaneous or induced labor, sonographic estimated fetal weight (EFW) recorded within 0–7 days before delivery, and complete data for the prespecified covariates used in the primary multivariable analysis. The 7-day window was selected as a pragmatic near-delivery interval to reduce discrepancy attributable to ongoing fetal growth while retaining the large majority of term records. Prelabor planned cesarean deliveries without documented spontaneous or induced labor were excluded, as were records with missing information required for cohort definition or the primary analysis.

No additional exclusions were applied on the basis of obstetric complications beyond the stated eligibility criteria. No major congenital anomaly was documented in the analytic cohort, and all deliveries included in the analytic cohort were live births. Specific indications for cesarean delivery and contraindications to vaginal delivery were not available in sufficiently standardized form for systematic classification.

### 2.2 Exposure variable

Sonographic fetal biometry included biparietal diameter (BPD), head circumference (HC), abdominal circumference (AC), and femur length (FL). Estimated fetal weight (EFW) was calculated using the Hadlock IV formula (11) and obtained from ultrasound reports in the clinical record. Ultrasound examinations were performed during routine obstetric care by multiple physician operators involved in obstetric and fetal-medicine ultrasound rather than by a single study-designated sonographer. Operators may have had access to previous ultrasound reports during routine clinical care. However, whether they reviewed earlier EFW measurements before the examination used in this analysis was not documented in the retrospective dataset.

EFW was the prenatal measure of interest and was analyzed on a 100-g scale. To express prenatal EFW relative to the newborn's actual birthweight, the EFW–birthweight discrepancy was calculated as follows:

EFW–birthweightdiscrepancy(g)=EFW(g)-neonatalbirthweight(g)

EFW–birthweight discrepancy was the main exposure in the primary regression model.

### 2.3 Study outcome

The primary outcome was cesarean delivery, coded as a binary variable. Because the analytic cohort was restricted to nulliparous women, all cesarean deliveries represented primary cesarean deliveries.

### 2.4 Adjustment variables

Prespecified covariates were neonatal birthweight, maternal age, gestational age at delivery, fetal sex, and scan-to-delivery interval. The model included both EFW–birthweight discrepancy and neonatal birthweight. Neonatal birthweight was included to estimate the association of a relatively higher prenatal EFW while accounting for eventual neonatal size. The other covariates were selected for their clinical relevance and consistent availability in the retrospective dataset.

### 2.5 Statistical analysis

Analyses were performed using R version 4.6.1. No *a priori* sample-size calculation was performed because this retrospective study included all available records meeting the eligibility criteria. Statistical precision was reported using 95% confidence intervals. Continuous variables are presented as mean ± standard deviation or median [interquartile range (IQR)], as appropriate. Categorical variables are presented as counts and percentages. For the unadjusted comparisons reported in Table 1, Welch's two-sample t-test was used for continuous variables presented as mean ± standard deviation, the Mann–Whitney U (Wilcoxon rank-sum) test was used for scan-to-delivery interval, and Pearson's chi-square test was used for categorical variables.

**Table 1:** Characteristics of the primary analytic cohort according to mode of delivery.

Characteristics	Overall (*N* = 353)	No cesarean delivery (*n* = 185)	Cesarean delivery (*n* = 168)	*P* value
Maternal age, years	28.00 ± 3.29	27.64 ± 3.27	28.41 ± 3.28	0.028
Gestational age at delivery, weeks	39.26 ± 0.78	39.18 ± 0.76	39.35 ± 0.79	0.032
Scan-to-delivery interval, days	2.0 [1.0–4.0]	2.0 [1.0–4.0]	2.0 [1.0–3.2]	0.447
Male fetus, n (%)	193 (54.7)	91 (49.2)	102 (60.7)	0.030
Labor induction, n (%)	48 (13.6)	17 (9.2)	31 (18.5)	0.011
EFW, g	3205 ± 267	3141 ± 273	3276 ± 242	<0.001
Neonatal birthweight, g	3178 ± 314	3116 ± 307	3246 ± 308	<0.001
EFW−neonatal birthweight, g	27 ± 199	25 ± 183	29 ± 215	0.842

Data are presented as mean ± standard deviation, median [IQR], or n (%). *P* values compare the no-cesarean and cesarean groups using appropriate unadjusted tests as described in the Methods. EFW, estimated fetal weight; IQR, interquartile range.

Pearson correlation was used to describe the linear relationship between EFW and neonatal birthweight. Agreement was assessed using signed error, absolute error, MAPE, the proportion of estimates within ±10% of birthweight, and Bland-Altman analysis (12). We reported the conventional mean bias and 95% limits of agreement and tested for proportional bias by regressing the EFW–birthweight difference on the mean of the two measurements. When proportional bias was present, regression-based 95% limits of agreement were calculated as the fitted difference ±1.96 times the residual standard deviation.

Because cesarean delivery was common in the cohort, we used modified Poisson regression with robust standard errors to estimate risk ratios directly rather than odds ratios (13). The primary model included EFW–birthweight discrepancy and neonatal birthweight, each per 100 g, with adjustment for maternal age, gestational age at delivery, fetal sex, and scan-to-delivery interval. Prespecified sensitivity analyses restricted the scan-to-delivery interval to ≤3 days, included only spontaneous labor, combined both restrictions, or limited neonatal birthweight to 2,500–3,999 g; an additional sensitivity analysis adjusted for induction of labor. Functional form was assessed using logistic regression models with natural cubic splines with 3 degrees of freedom, compared with corresponding linear terms using likelihood-ratio tests. Quadratic terms were evaluated separately in modified Poisson models. Multicollinearity was assessed using variance inflation factors and the condition number.

Complete-case analysis was performed without imputation. Overall missingness immediately before complete-case restriction was low (21/794 records, 2.6%). Therefore, this approach was retained. Variable-specific missingness is reported in Supplementary Table S2. All tests were two-sided, with *α*=0.05.

### 2.6 Ethics approval statement

The study was approved by the Institutional Review Board for Biomedical Research of Vinmec Times City International Hospital (approval no. 145/2025/CN/HĐĐĐ VMEC). The requirement for informed consent was waived for this retrospective study using deidentified data.

## 3 Results

### 3.1 Study population

Among 930 unique records available for eligibility assessment, 879 were term deliveries. After restricting to a scan-to-delivery interval of 0–7 days and complete main variables, 773 records remained. 353 women met the final eligibility criteria for the primary analytic cohort (Supplementary Figure S1). Of these, 168 (47.6%) underwent cesarean delivery. Variable-specific missingness is shown in Supplementary Table S2.

Mean maternal age was 28.00 ± 3.29 years and mean gestational age at delivery was 39.26 ± 0.78 weeks. Mean EFW was 3,205 ± 267 g and mean neonatal birthweight was 3,178 ± 314 g. Baseline characteristics and unadjusted between-group comparisons are shown in Table 1.

### 3.2 Agreement between EFW and neonatal birthweight

Prenatal EFW and neonatal birthweight were positively correlated (Pearson r = 0.78). Mean signed error between EFW and neonatal birthweight was +27.1 g, and MAPE was 5.15%. Overall, 314 of 353 estimates (89.0%) were within ±10% of neonatal birthweight (Table 2).

**Table 2:** Agreement between sonographic estimated fetal weight and neonatal birthweight.

Measure	Overall (*N* = 353)	Q1 (*n* = 89)	Q2 (*n* = 88)	Q3 (*n* = 88)	Q4 (*n* = 88)
Median neonatal birthweight, g	-	2800	3070	3290	3545
Mean error EFW−BW, g	+27.1	+144.2	+78.9	+15.9	−132.0
Mean error, %	+1.19	+5.26	+2.60	+0.49	−3.64
MAPE, %	5.15	6.36	5.29	3.97	4.95
EFW within ±10% BW, n (%)	314 (89.0)	68 (76.4)	78 (88.6)	86 (97.7)	82 (93.2)

Quartiles were defined according to the distribution of neonatal birthweight. EFW, estimated fetal weight; BW, birthweight; MAPE, mean absolute percentage error.

The conventional Bland-Altman 95% limits of agreement were −362.6 to +416.8 g. Bland-Altman regression showed significant proportional bias. The EFW–birthweight difference decreased by 18.2 g for each 100-g increase in mean weight (95% CI −25.6 to −10.8; *p* < 0.001). Accordingly, EFW tended to overestimate birthweight at lower weights and underestimate birthweight at higher weights. Figure 1 displays the regression-based bias and limits across the observed weight range.

**Figure 1:**
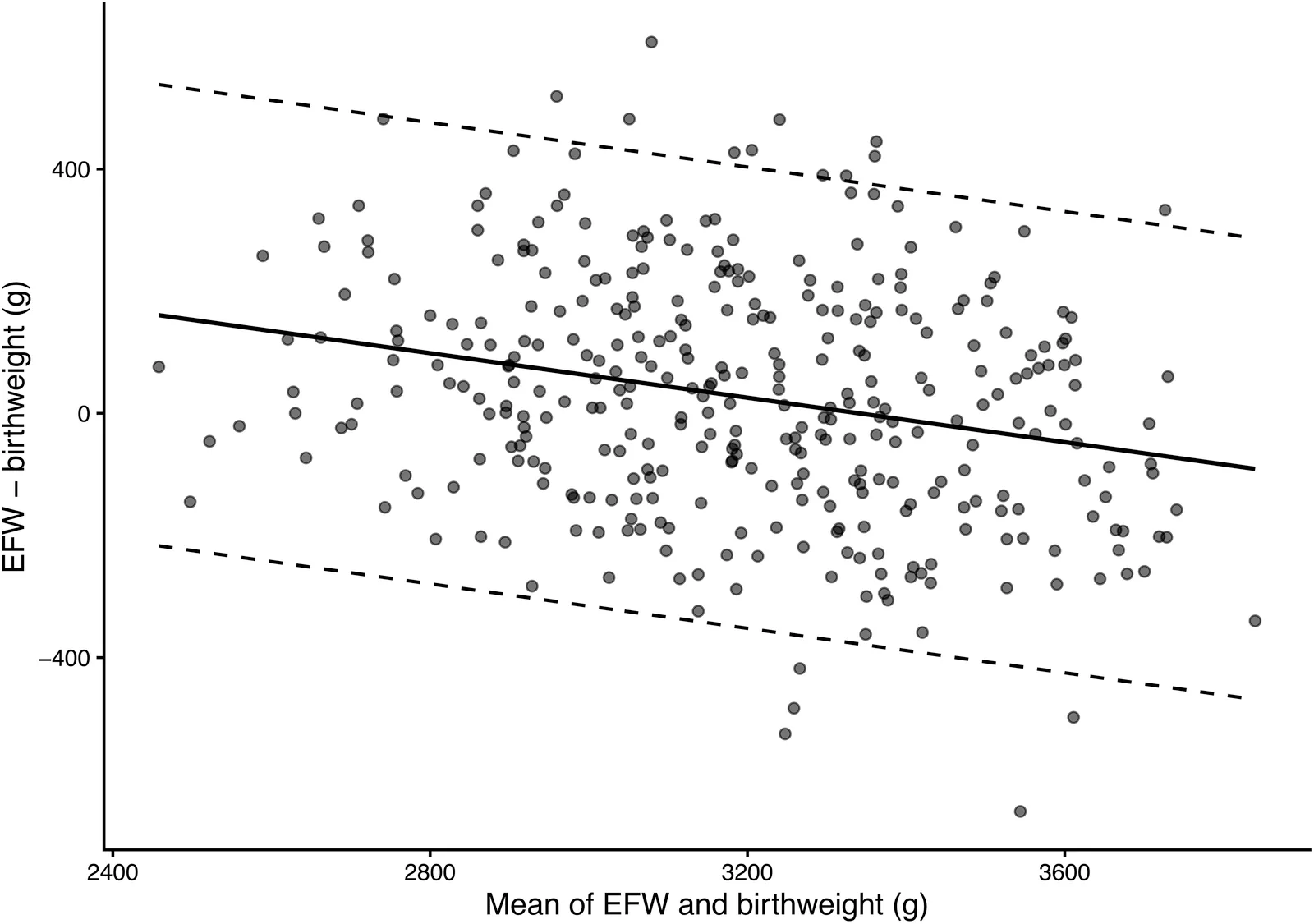
Bland–Altman plot of agreement between sonographic estimated fetal weight and neonatal birthweight (*N* = 353). The solid line shows the regression-based mean difference and the dashed lines show the corresponding 95% limits of agreement. EFW, estimated fetal weight.

### 3.3 Association between EFW and cesarean delivery

In the primary model, each 100-g greater EFW–birthweight discrepancy was associated with an 11% higher risk of cesarean delivery after accounting for neonatal birthweight and adjusting for maternal age, gestational age at delivery, fetal sex, and scan-to-delivery interval (aRR 1.11, 95% CI 1.04–1.20; *p* = 0.003) (Table 3).

**Table 3:** Association of EFW–birthweight discrepancy with primary cesarean delivery.

Variable	aRR	95% CI	*P* value
EFW–birthweight discrepancy, per +100 g	1.11	1.04–1.20	0.003
Neonatal birthweight, per +100 g	1.11	1.05–1.16	<0.001
Maternal age, per +1 year	1.04	1.00–1.07	0.029
Gestational age at delivery, per +1 week	1.04	0.88–1.22	0.671
Male fetus	1.28	1.02–1.61	0.035
Scan-to-delivery interval, per +1 day	1.03	0.97–1.09	0.359

Adjusted risk ratios were estimated using modified Poisson regression with robust standard errors. The model included EFW–birthweight discrepancy, neonatal birthweight, maternal age, gestational age at delivery, fetal sex, and scan-to-delivery interval. EFW, estimated fetal weight; aRR, adjusted risk ratio; CI, confidence interval.

The association was consistent across the prespecified sensitivity analyses, with adjusted risk ratios ranging from 1.11 to 1.14 (Figure 2; Supplementary Table S1). Additional adjustment for induction of labor produced an essentially unchanged estimate (aRR 1.11, 95% CI 1.03–1.19; *p* = 0.004).

**Figure 2:**
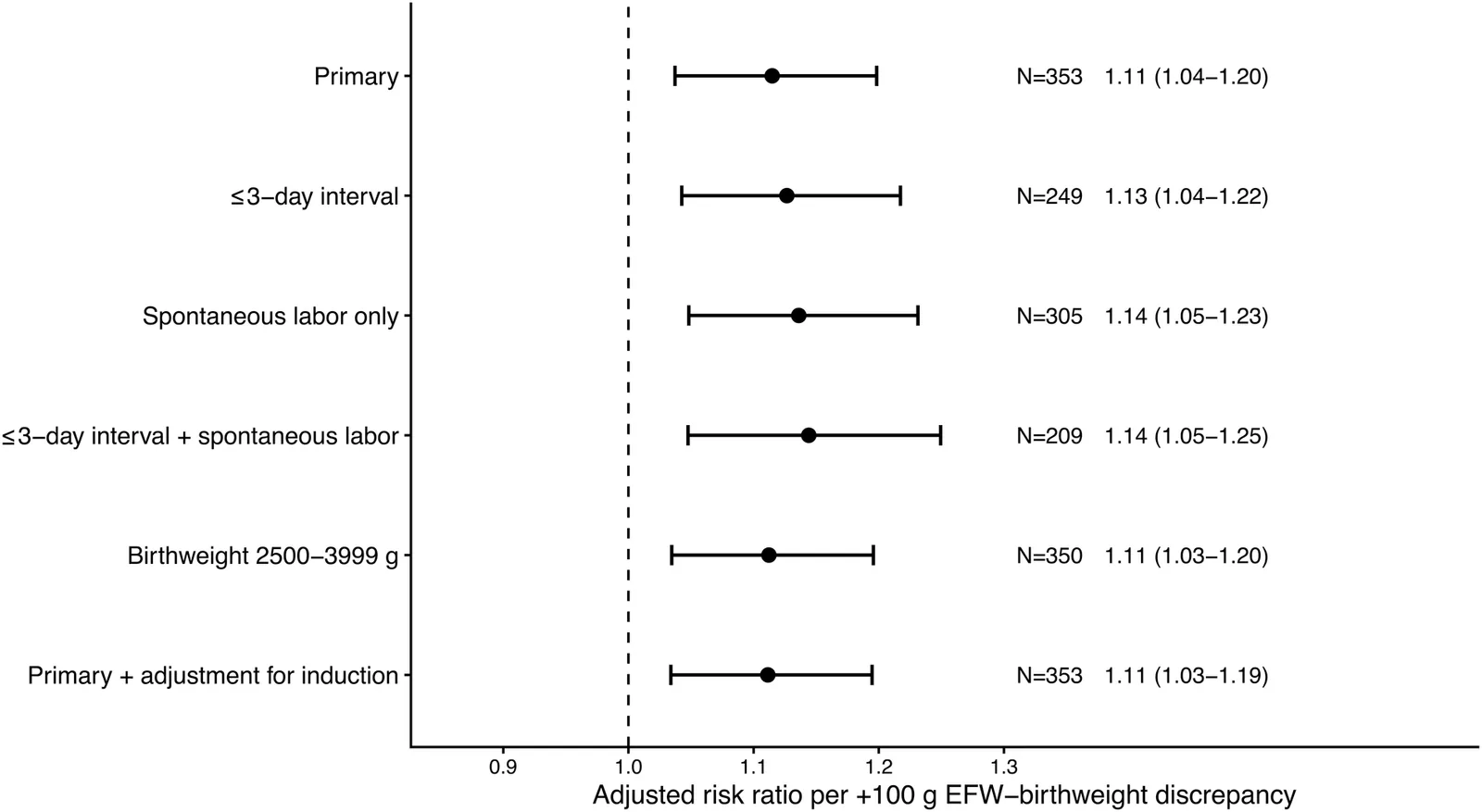
Primary and sensitivity analyses of the association between EFW–birthweight discrepancy and primary cesarean delivery. Estimates are adjusted risk ratios per 100-g increase in EFW–birthweight discrepancy. Sensitivity analyses used overlapping subsets of the primary cohort. The induction-adjusted analysis used the full primary cohort. EFW, estimated fetal weight.

Secondary analyses showed some evidence that the association was not fully linear (spline *p* = 0.043; quadratic *p* = 0.016). The spline-based association is illustrated in Supplementary Figure S2. There was no evidence of nonlinearity for neonatal birthweight (*p* = 0.870). No important multicollinearity was detected in the primary model.

## 4 Discussion

In this retrospective cohort of 353 term nulliparous women, a relatively higher prenatal EFW compared with eventual neonatal birthweight was associated with a higher risk of cesarean delivery after accounting for neonatal birthweight and prespecified covariates. The primary linear model estimated an approximately 11% higher risk per 100-g greater EFW–birthweight discrepancy. This should be interpreted as an average association after accounting for neonatal birthweight, not as evidence of a causal effect. The models were used to estimate associations and were not developed or validated for individual clinical prediction.

Our findings are consistent with previous studies linking sonographic EFW or prenatal suspicion of a large fetus with cesarean delivery. Little et al. found that having a sonographic EFW recorded within one month before delivery was associated with higher odds of cesarean delivery even after adjustment including neonatal birthweight (14). In a large multicenter cohort of women attempting vaginal delivery at term, Froehlich et al. similarly found that documented ultrasound EFW remained associated with cesarean delivery after adjustment including actual birthweight and other potential confounders (15). Among term nulliparous women undergoing induction of labor, Blackwell et al. found that ultrasound EFW overestimation by ≥15% relative to actual birthweight was associated with an increased risk of cesarean delivery for labor arrest (16). Findings have not been entirely consistent, however, as Yee et al. found no significant increase in cesarean delivery among term nulliparous women delivering neonates >3,500 g who underwent late-third-trimester growth sonography compared with those who did not (17).

More recent evidence further supports an association between prenatal assessment of fetal size and cesarean delivery. Cooley et al. found that an ultrasound diagnosis of LGA was associated with a higher likelihood of cesarean delivery, including among nulliparous, term, singleton, cephalic pregnancies, despite a positive predictive value of only approximately 50% (9). Sgayer et al. similarly showed that even among newborns with an actual birthweight ≥4,000 g, cesarean delivery was more frequent when macrosomia had been suspected prenatally (10). More recently, Lopian et al. reported that pregnancies screening positive for LGA or macrosomia were less likely to undergo a trial of labor and had a higher risk of intrapartum cesarean delivery, including false-positive cases (6). Unlike studies that mainly classified fetuses as LGA or macrosomic, we analyzed EFW as a continuous measure and accounted for actual neonatal birthweight.

One possible pathway, which could not be directly evaluated in the present dataset, is that knowledge of a relatively high EFW may influence clinicians’ perception of obstetric risk, including concern about cephalopelvic disproportion, labor dystocia, shoulder dystocia, or neonatal injury (7). Previous studies reporting increased intervention following prenatal suspicion of LGA or macrosomia are compatible with such a labeling pathway (6, 9, 10). However, our findings do not establish this mechanism because clinician awareness of EFW, counseling, labor-management decisions, and specific indications for cesarean delivery were not systematically recorded.

The clinical relevance of this association also depends on the accuracy of EFW. In our cohort, average EFW accuracy was relatively good, with a mean bias of +27.1 g and 89.0% of estimates within ±10% of neonatal birthweight. However, agreement was not uniform across the weight range. Bland-Altman regression showed proportional bias, with the EFW–birthweight difference decreasing by approximately 18 g for every 100-g increase in mean weight. This pattern was consistent with the quartile analysis, in which EFW tended to overestimate lower birthweights and underestimate higher birthweights. These overall limits do not mean that EFW was equally accurate for every fetus.

Across the prespecified sensitivity analyses, estimates remained similar, with aRRs ranging from 1.11 to 1.14. Additional adjustment for induction of labor also produced an essentially unchanged estimate. Because prenatal EFW may influence the decision to induce labor, induction was not included in the primary model. Adding it in a sensitivity analysis did not materially change the result. The association was not perfectly linear. Therefore, the 11% increase per 100 g should be viewed as an average across the observed range rather than as an identical increase at every level of discrepancy.

The cesarean delivery rate in this cohort (47.6%) was relatively high but consistent with reports from Vietnam, where rates of approximately 40%–50% have been described in some obstetric populations and a 47.1% rate was reported among primiparous women in a survey conducted in two Vietnamese cities (18). Local practice patterns and nonmedical factors may also contribute to mode-of-delivery decisions (19). The present findings should therefore be interpreted within the Vietnamese obstetric context, and the magnitude of the observed association may not be directly generalizable to settings with substantially lower cesarean rates or different thresholds for induction, intrapartum intervention, and cesarean delivery.

This study has several strengths. Restriction to term nulliparous women with singleton, cephalic pregnancies reduced clinical heterogeneity. The near-delivery ultrasound window limited discrepancy attributable to ongoing fetal growth, and the ≤3-day sensitivity analysis yielded similar results. Analyzing EFW as a continuous measure avoided arbitrary categories. Accounting for neonatal birthweight allowed us to examine whether a relatively higher prenatal EFW was associated with cesarean delivery beyond the effect of actual neonatal size. We also evaluated EFW–birthweight agreement using complementary metrics and examined robustness across multiple sensitivity analyses, including additional adjustment for induction of labor.

Several limitations should be considered. First, the retrospective single-center design precludes causal inference and limits external generalizability. Second, several clinically important potential confounders were unavailable in sufficiently standardized form for multivariable adjustment, including maternal body mass index, diabetes status, gestational weight gain, maternal height, cervical findings, labor progress, epidural analgesia, indication for induction, and indication for cesarean delivery. Maternal obesity and gestational diabetes have been associated with a higher likelihood of cesarean delivery (20, 21). An unfavorable cervical status before induction is associated with a higher likelihood of cesarean delivery, while abnormal labor progress and labor arrest are important determinants of intrapartum management and cesarean delivery decisions (22, 23). Because these variables were not available in our dataset, residual confounding remains possible. These unmeasured factors could affect both cesarean risk and the relationship between EFW and actual birthweight. Depending on these relationships, residual confounding could either strengthen or weaken the observed association, so its direction cannot be determined from our data. No major congenital anomaly was documented in the analytic cohort. However, the dataset did not contain standardized variables sufficient to classify all obstetric complications or contraindications to vaginal delivery. Third, ultrasound examinations were performed by multiple clinicians, and interobserver variability in biometric acquisition was not formally assessed. Differences between operators may have added variability to the EFW measurements and may have weakened or altered the observed association. Fourth, we could not determine systematically whether clinicians were aware of the recorded EFW when delivery decisions were made or whether EFW influenced counseling or intervention thresholds. The observed association therefore cannot establish a causal labeling or decision-making effect. Fifth, neonatal birthweight was measured after ultrasound and is not identical to fetal weight at the time of scanning. Sixth, the missing completely at random (MCAR) assumption could not be verified from the available data. Although few records were excluded because of missing data, selection bias from nonrandom missingness cannot be ruled out. Finally, EFW showed substantial individual-level variability and weight-dependent proportional bias despite relatively good average accuracy.

Clinically, the observed association should not be interpreted as proof that knowledge of EFW causes cesarean delivery. Rather, EFW should be interpreted alongside labor progress and other maternal and fetal factors, with recognition that individual estimates remain imprecise (3, 5). Prospective studies that record the indication for near-delivery ultrasound, clinician awareness of EFW, counseling, and the timing and indication for cesarean delivery are needed to distinguish a potential labeling effect from residual confounding and other pathways.

## 5 Conclusion

Among term nulliparous women, a relatively higher prenatal sonographic EFW compared with eventual neonatal birthweight was associated with a higher risk of primary cesarean delivery. The primary linear model estimated an average 11% higher risk per 100-g greater EFW–birthweight discrepancy. These findings do not show that knowledge of EFW caused cesarean delivery and should be interpreted in light of residual confounding and the limited accuracy of EFW in individual pregnancies.

## Data Availability

The data analyzed in this study is subject to the following licenses/restrictions: The datasets presented in this article are not publicly available because they contain patient-level clinical data subject to privacy and ethical restrictions. Requests to access these datasets should be directed to Quang-Chien Chu, cqchien.med@gmail.com.
