# Supplementary material for: Association of sonographic estimated fetal weight with primary cesarean delivery after adjustment for neonatal birthweight in term nulliparous pregnancies
Source: Front Med (Lausanne). 2026 Oct 22;13:1989181. doi: 10.3389/fmed.2026.1989181 (PMC13736597; doi:10.3389/fmed.2026.1989181)
Supplement: Supplementary file 1 [file Datasheet1.pdf]

Supplementary Material

1 Supplementary Figures

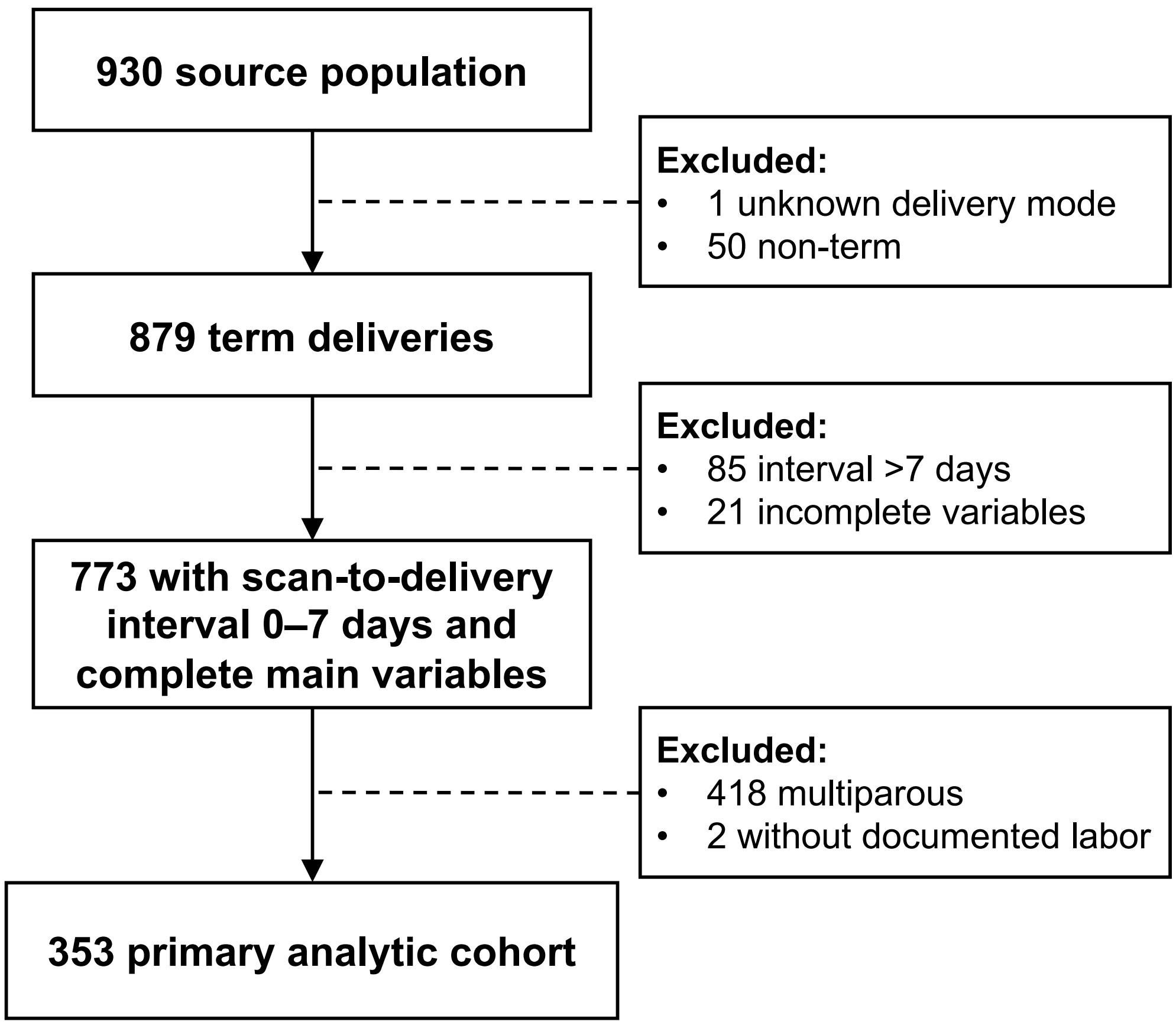

Supplementary Figure S1. Sequential derivation of the primary analytic cohort from the initial source population.

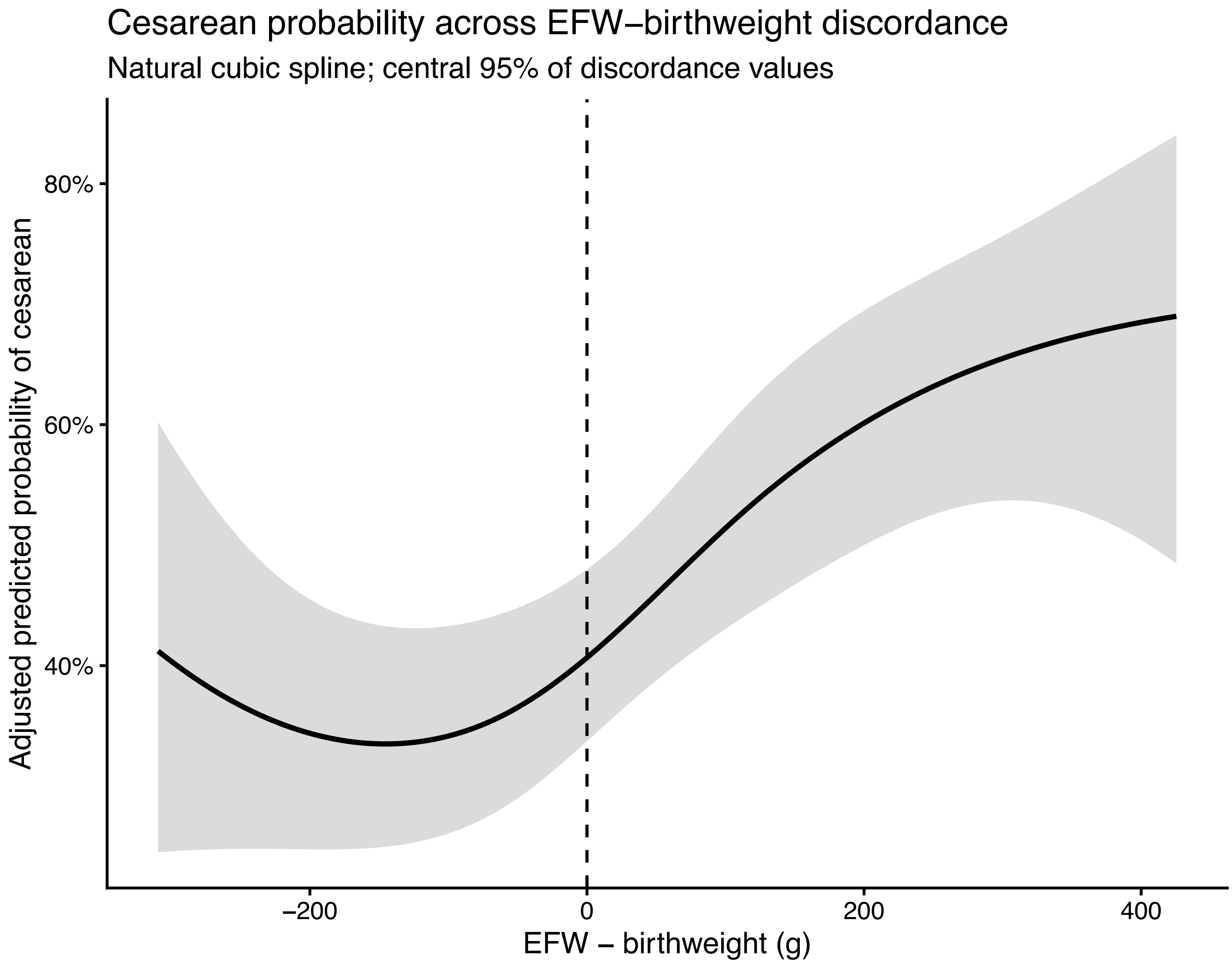

Supplementary Figure S2. Flexible modeling of EFW–birthweight discrepancy and cesarean delivery. The solid line shows the adjusted predicted probability of cesarean delivery from a logistic regression model using a natural cubic spline with 3 degrees of freedom across the central 95% of observed discrepancy values; the shaded band represents the 95% confidence interval and the vertical dashed line indicates zero discrepancy. There was modest evidence of nonlinearity compared with a linear term (likelihood-ratio p=0.043). The spline was used to assess functional form; the modified Poisson model remained the primary inferential model.

2 Supplementary Tables

Supplementary Table S1. Sensitivity analyses of the primary association.

Analysis	N	Cesarean, n	aRR per +100 g discrepancy	95% CI	P value
Primary cohort	353	168	1.11	1.04–1.20	0.003
Spontaneous labor only	305	137	1.14	1.05–1.23	0.002
Scan-to-delivery interval 0–3 days	249	126	1.13	1.04–1.22	0.003
0–3 days + spontaneous labor	209	102	1.14	1.05–1.25	0.003
Birthweight 2500–3999 g	350	167	1.11	1.03–1.20	0.004
Additional adjustment for induction of labor	353	168	1.11	1.03–1.19	0.004

All effect estimates represent the adjusted risk ratio per 100-g increase in EFW–birthweight discrepancy. Primary and subgroup models adjusted for neonatal birthweight, maternal age, gestational age at delivery, fetal sex, and scan-to-delivery interval. The induction-adjusted analysis additionally included induction of labor. Subgroup analyses use overlapping subsets of the primary cohort and are not independent. EFW, estimated fetal weight; aRR, adjusted risk ratio; CI, confidence interval.

Supplementary Table S2. Variable-specific missingness before complete-case restriction.

Variable	Missing, n	Denominator	Missing, %
Cesarean delivery	0	794	0.00
Nulliparity/parity status	7	794	0.88
Gestational age at delivery	0	794	0.00
Scan-to-delivery interval	0	794	0.00
Neonatal birthweight	0	794	0.00
Estimated fetal weight	0	794	0.00
Maternal age	13	794	1.64
Fetal sex	1	794	0.13
Labor onset	0	794	0.00

Missingness is shown among the 794 records that met the term-delivery and 0–7-day scan-to-delivery criteria, immediately before complete-case restriction. In total, 21/794 records (2.6%) were excluded because at least one item required for cohort definition or the primary analysis was incomplete. Complete-case analysis was retained because overall missingness was low.
